# Ancestral polymorphism and recent invasion of transposable elements in *Drosophila* species

**DOI:** 10.1186/1471-2148-12-119

**Published:** 2012-07-23

**Authors:** Elaine Silva Dias, Claudia Marcia Aparecida Carareto

**Affiliations:** 1 Department of Biology, São José do Rio Preto, UNESP - São Paulo State University, São Paulo, Brazil

**Keywords:** Transposable elements, Ancestral polymorphism, Horizontal transfer, Introgressive hybridization, Recent invasion, *Drosophila melanogaster* group

## Abstract

**Background:**

During the evolution of transposable elements, some processes, such as ancestral polymorphisms and horizontal transfer of sequences between species, can produce incongruences in phylogenies. We investigated the evolutionary history of the transposable elements *Bari* and *412* in the sequenced genomes of the *Drosophila melanogaster* group and in the sibling species *D. melanogaster* and *D. simulans* using traditional phylogenetic and network approaches.

**Results:**

Maximum likelihood (ML) phylogenetic analyses revealed incongruences and unresolved relationships for both the *Bari* and *412* elements. The DNA transposon *Bari* within the *D. ananassae* genome is more closely related to the element of the *melanogaster* complex than to the sequence in *D. erecta*, which is inconsistent with the species phylogeny. Divergence analysis and the comparison of the rate of synonymous substitutions per synonymous site of the *Bari* and host gene sequences explain the incongruence as an ancestral polymorphism that was inherited stochastically by the derived species. Unresolved relationships were observed in the ML phylogeny of both elements involving *D. melanogaster*, *D. simulans* and *D. sechellia*. A network approach was used to attempt to resolve these relationships. The resulting tree suggests recent transfers of both elements between *D. melanogaster* and *D. simulans*. The divergence values of the elements between these species support this conclusion.

**Conclusions:**

We showed that ancestral polymorphism and recent invasion of genomes due to introgression or horizontal transfer between species occurred during the evolutionary history of the *Bari* and *412* elements in the *melanogaster* group. These invasions likely occurred in Africa during the Pleistocene, before the worldwide expansion of *D. melanogaster* and *D. simulans*.

## Background

Transposable elements are segments of repetitive DNA that can mobilize and propagate within host genomes. They have long been considered to be selfish DNA sequences because of the deleterious effects of their mobilization on the host genome. Recent advances in genome analysis methods have revealed the significant contribution of transposable elements to genome evolution as sources of genomic novelty, as they can promote rearrangements [1] and duplications [2] and can produce new regulatory sequences [3] that drive the changes necessary for genome evolution [4]. The emergence of transposable elements in a genome can occur in three ways: *de novo* emergence, by the recombination of existing elements within genomes; horizontal transfer, by a vector; and introgression, by hybridization between two species (one with and one without a given element) [5,6]. The origin of a transposable element in a new genome by the last two processes may produce incongruences when the phylogeny of the elements is compared to those of the species that harbor them. In addition, incongruence can also be produced when two or more variants in an ancestral lineage are stochastically inherited by the derived lineages. Horizontal transfer has been reported in several organisms (for a review see [7,8]), primarily between closely related species, given the requirement of shared time and space. In many cases, such species also share putative vectors (for a review see [9]); however, the occurrence of phylogenetic incongruence due to the stochastic inheritance of ancestral polymorphisms, although potentially common and frequently given as an alternative hypothesis to horizontal transfers [8], is less often demonstrated in the literature.

The genus *Drosophila* has been the focus of numerous studies involving transposable elements, and the aforementioned processes have been described in these species via bioinformatics analyses and analysis of natural populations [8,9]; such studies have focused on species of the *melanogaster* group, especially the *melanogaster* subgroup. This subgroup comprises nine species (*D. yakuba*, *D. teissieri*, *D. santomea*, *D. erecta*, *D. orena*, *D. melanogaster*, *D. simulans*, *D. sechellia* and *D. mauritiana*) that differ in many aspects, such as geographical distribution and food and host preference, but that diverged relatively recently. The subgroup is one of ten subgroups of *melanogaster*, eight of which are found in Asia and three in Africa (*melanogaster*, *montium* and *ananassae*); however, only the *melanogaster* subgroup is endemic to the Afrotropical region [10]. This subgroup is thought to have originated from a proto-*melanogaster* founder population that arrived in Africa 17–20 Mya from the Oriental region. This founder population gave rise to the evolutionary lineages that produced the *erecta* supercomplex approximately 13–15 Mya, the *yakuba* complex approximately 8–15 Mya and, more recently, the basal lineage of the *melanogaster* supercomplex. Within this supercomplex, the most basal species, *D. melanogaster*, arose between 2 and 3 Mya; *D. simulans, D. sechellia* and *D. mauritiana* emerged very recently, no more than 0.5 Mya [11,12]. *D. melanogaster* and *D. simulans* are widespread due to very recent global colonization. Also widespread is *D. ananassae*; this species belongs to the *ananassae* subgroup, the basal clade in the *melanogaster* species group [13,14] (Species Phylogeny, Additional file 1 Figure S1). This species originated in southeast Asia and subsequently dispersed to other parts of the world, possibly through human activity [15]. The availability of the complete genomes of five species in the subgroup [16] enables the description of numerous transposable element transfers [9]. Meanwhile, the sequencing of just one strain’s genome (in four of the five species) and the variable rates of genome coverage can prevent an accurate understanding of the evolutionary history of the elements in these species. One potentially important species is *D. ananassae*, whose genome is available but rarely included in studies. Its widespread distribution, from tropical to subtropical regions, and highly substructured populations make *D. ananassae * a model for studies of genetic variation [17], such as the characterization of transposable elements.

Among the transposable elements studied in the *melanogaster* subgroup regarding horizontal transmission or introgression are the DNA transposon *Bari* (transfer between *D. melanogaster* and *D. simulans *[18]), and the retrotransposon *412* (transfers between *D. melanogaster*, *D. simulans* and *D. sechellia *[19,20]). *Bari*, a DNA transposon belonging to the *Tc1-Mariner* superfamily, is an ancient element in the evolutionary lineage of drosophilids that is widespread in both the *Drosophila* and *Sophophora* subgenera of the *Drosophila* genus, although it seems to have been lost in some species [21,22]. Within the genus, there are interspecific structural variations in the terminal-inverted repeats (TIRs), the size of which would have changed over time [23]. Some variants, such as *Bari2* (distributed in both *Drosophila* and *Sophophora* species) and *Bari3* (described in *D. willistoni*, *D. pseudoobscura* and *D. mojavensis*) harbor long TIRs, called LIRs (Long Inverted Repeats), which are approximately 250 bp long. Others, such as *Bari1*, which is present in the *melanogaster* complex only, contain short TIRs, called SIRs (Short Inverted Repeats), which are approximately 26-bp long. These three variants, which share over 50% amino-acid similarity, characterize three subfamilies derived from a common *Bari*-element ancestor [23]. The element *412*, a LTR (Long Terminal Repeats) retrotransposon that belongs to a *Gypsy*-like superfamily, also seems to have appeared early in the evolution of the Drosophilidae family and to have been subsequently lost in some lineages [24]. In contrast to *D. melanogaster*, the genome of which contains only one *412-*subfamily element, *D. simulans* has two intragenomic variants that differ in the size of the 5´LTR – UTR regulatory region. These two subfamilies arose from rearrangements and insertion-deletion events that produced new elements that may be capable of escaping host control [24].

Here, we studied the occurrence of both *Bari* and *412* in the six sequenced genomes of the *melanogaster* group (*D. ananassae, D. erecta*, *D. yakuba*, *D. melanogaster*, *D. simulans* and *D. sechellia*). In addition, we compared the *in silico* results with those obtained from geographic strains of the sibling species *D. melanogaster* and *D. simulans* to uncover the evolutionary history of these transposable elements in the subgroup. The results will expand our understanding of the processes that shaped their evolution. We showed that at least two *Bari* variants were present in the ancestral lineage of the *melanogaster* group and were stochastically inherited, leading to incongruences between the phylogeny of the species and that of the transposable element. We also showed that the transfer of *Bari* and *412* elements between *D. simulans* and *D. melanogaster* occurred before the worldwide dispersal of both species and involved only one sequence per element. Thus, ancestral polymorphism, losses and reintroductions can explain the evolutionary distribution of these elements in these species of the *melanogaster* species subgroup.

## Results

Using the deposited sequences and the reference sequences for the transposable elements *Bari* and *412*, we searched in the sequenced genomes of the *melanogaster* group. Homologous full-length and fragmentary sequences of both elements were found in all species (see Table S1 in Additional File 2 and Table S5 in Additional File 3). The fragments were not included in the analyses because most of them contained large deletions and many nucleotide substitutions, which prevent the estimation of the *Ks* values and the corresponding time of divergence between the sequences. Therefore, only the full-length sequences, with both TIRs for *Bari* and both LTRs for *412*, were used for the analyses.

### DNA transposon Bari

**Figure 1:**
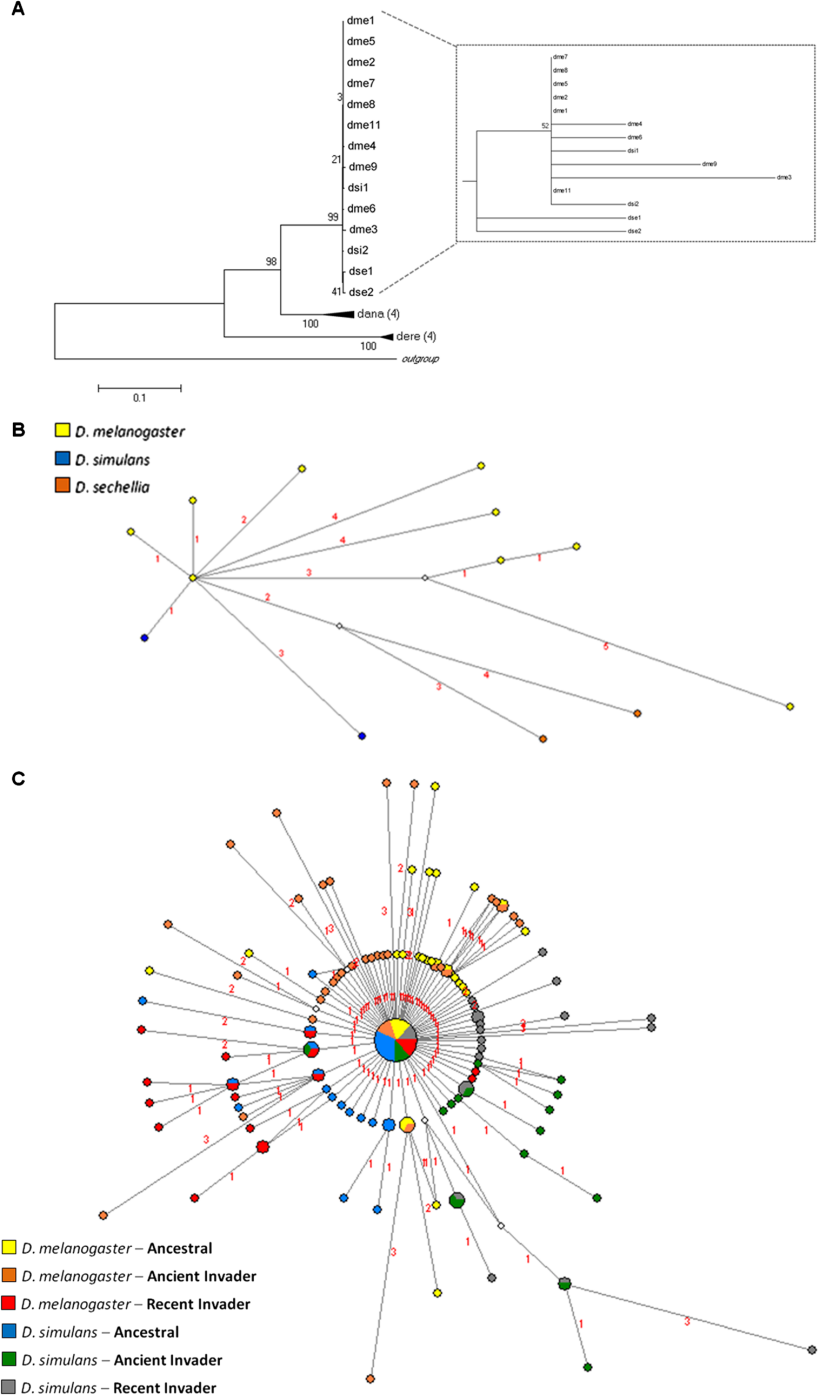
**Phylogenetic reconstructions conducted using sequences of the DNA transposon *Bari *in the *melanogaster *group of *Drosophila*.** (**A**) Phylogenetic analysis by maximum likelihood and (**B**) Network using the sequences of the transposase of the full-length copies obtained from sequenced genomes of the *melanogaster* group; (**C**) Network reconstructed using a region of the transposase sequenced in natural populations of the *D. melanogaster* and *D. simulans* species. In the network, full circles correspond to the sampled sequences; empty circles correspond to median vectors ancestral nodes, which represent lost sequences or sequences not sampled. Circle size corresponds to sequence frequency; branch size is proportional to the number of mutations that occurred, as indicated by the numbers above the branches.

The number of full-length *Bari* sequences varied by species: 11 were found in *D. melanogaster*, two were found in both *D. simulans* and *D. sechellia*, seven in *D. erecta* and four in *D. ananassae* (see Table S2 in Additional File 2). The only sequence found in *D. yakuba* was a 215 bp fragment resembling the *Bari* of *D. erecta*. The ML reconstruction of evolutionary relationships among the full-length sequences is shown in Figure 1A and Additional file 2 Figure S2. The sequences of *D. ananassae* and *D. erecta* are clustered in well-supported monophyletic clades. Also well-supported is the clade grouping the sequences of the *melanogaster* complex (*D. melanogaster*, *D. simulans* and *D. sechellia*), albeit in a polytomic branch; however, the *Bari* sequences of *D. ananassae* cluster more closely to those of the *melanogaster* complex than to those of *D. erecta*, which is inconsistent with the species phylogeny (Additional File 1). The *K2p* distances further contribute to this incongruence, with the sequences of the *melanogaster* complex and *D. ananassae* being less divergent from each other (*melanogaster* complex vs. *D. ananassae* = 0.171 ± 0.012; *melanogaster* complex vs. *D. erecta* = 0.372 ± 0.024; *D. ananassae* vs. *D. erecta* = 0.380 ± 0.023; see Table S3 in Additional File 2).

Two processes could be responsible for the phylogenetic incongruence observed in the *Bari* phylogeny: recent invasion of the *melanogaster* complex by a *Bari* sequence from *D. ananassae* (or vice versa) or the existence of an ancestral polymorphism followed by stochastic inheritance. To distinguish between these two possibilities, we estimated *Ks*, the rate of synonymous substitutions per synonymous site, which provide a measure of divergence in neutral sites, and the time of divergence of the *Bari* sequences and of the host genes (i.e., ADH and GAPDH). If the incongruence in the phylogeny is due to differential fixation of *Bari* variants in the common ancestor and is evolving vertically, then the *Ks* values of *Bari* and of the host genes and their time of divergence should be equivalent; however, if the estimates for the transposable element sequences are significantly lower than those for the host genes, then *Bari* was likely transferred after the species divergence, through horizontal transfer or species hybridization. The sequences of *D. sechellia* and *D. erecta* were not utilized in this analysis because they contained large numbers of stop codons and small deletions. The *Bari* sequences of *D. ananassae* showed few premature stop codons, all of which were excluded from the alignment, so these sequences were used in the *Ks* estimate. The average *Ks* values of the *Bari* sequences and host genes were as follows: *D. melanogaster* vs. *D. ananassae*, *Bari Ks* = 0.409 ± 0.049 and host genes *Ks* = 0.428 ± 0.058; *D. simulans* vs. *D. ananassae*, *Bari Ks* = 0.409 ± 0.047 and host genes *Ks* = 0.422 ± 0.055 (see Tables S4 and S5 in Additional File 2). Using the estimated *Ks* and 0.011 substitutions per site per million years (My) [25] as the rate of synonymous substitution, the average time of divergence of *Bari* in these species was estimated at 17.68 My, and that of the host genes was 19.34 My. During this latter period, the lineages that gave rise to *D. ananassae* and the *melanogaster* subgroup were still evolving in Asia [10], suggesting that the *Bari* sequences diverged from a common sequence in the common ancestor. These estimates suggest that the incongruence resulted from stochastic retention of the same *Bari1*-like variant by the ancestors of *D. ananassae* and of the *melanogaster* complex. The loss of parts of the TIRs followed, yielding long TIRs in *D. ananassae* and short TIRs in the *melanogaster* complex [23].

The ML tree did not allow us to resolve the relationships among the *Bari1* sequences within the *melanogaster* complex (*D. melanogaster*, *D. simulans*, *D. sechellia*) because the sequences are very similar and cluster within an unresolved branch. We therefore reconstructed the sequence relationships using a network tree. This approach can resolve relationships among sequences with low diversity and can thus be used to infer the origin of multiple copies from a unique sequence. In addition, the network reveals relationships between ancestral and derived sequences and introduces median vectors, which represent ancestral, lost or unsampled sequences [26,27]; these relationships cannot be inferred from the classical phylogenies.

The network shows a second phylogenetic incongruence in the *Bari1* sequences, revealing a closer relationship between the copies of *D. simulans* and *D. melanogaster* than either has to *D. sechellia* (Figure 1B). The two full-length sequences of *D. simulans* are directly related to a unique sequence of *D. melanogaster*, which is centrally positioned on the network and is the sequence from which all the other sequences of this species diverged. Moreover, all sequences of *D. melanogaster* are very similar (*K2p* = 0.0020 ± 0.0006), suggesting a recent origin. The age of these copies was estimated at ~ 40,000 y (*Ks* = 0.00086 ± 0.00084), and the longest time of divergence between the *Bari1* sequences of *D. melanogaster* and that of *D. simulans* was estimated at ~ 196,900 y (*Ks* = 0.0004 ± 0.0004; average time = 32,800 y; shortest time = 0; see Table S4 in Additional File 2). Because the *D. melanogaster* and *D. simulans* lineages split from a common ancestor between 2 and 3 Mya [11,12], the network topology and the divergence time of the *Bari1* sequences in both species are inconsistent with the species phylogeny and the estimated divergence time. These data suggest a very recent transfer of *Bari1* from *D. simulans* to *D. melanogaster* and further suggest that following this transfer, the sequence remained active and dispersed within the *D. melanogaster* genome, producing the similar copies that are observed today. The short branches and the presence of several similar copies in the *D. melanogaster* network, all derived from the same sequence, are evidence of transposition burst, a common process after the introduction of a new element into a naïve genome [20], supporting the hypothesis of the transfer from *D. simulans* to *D. melanogaster*.

**Figure 2:**
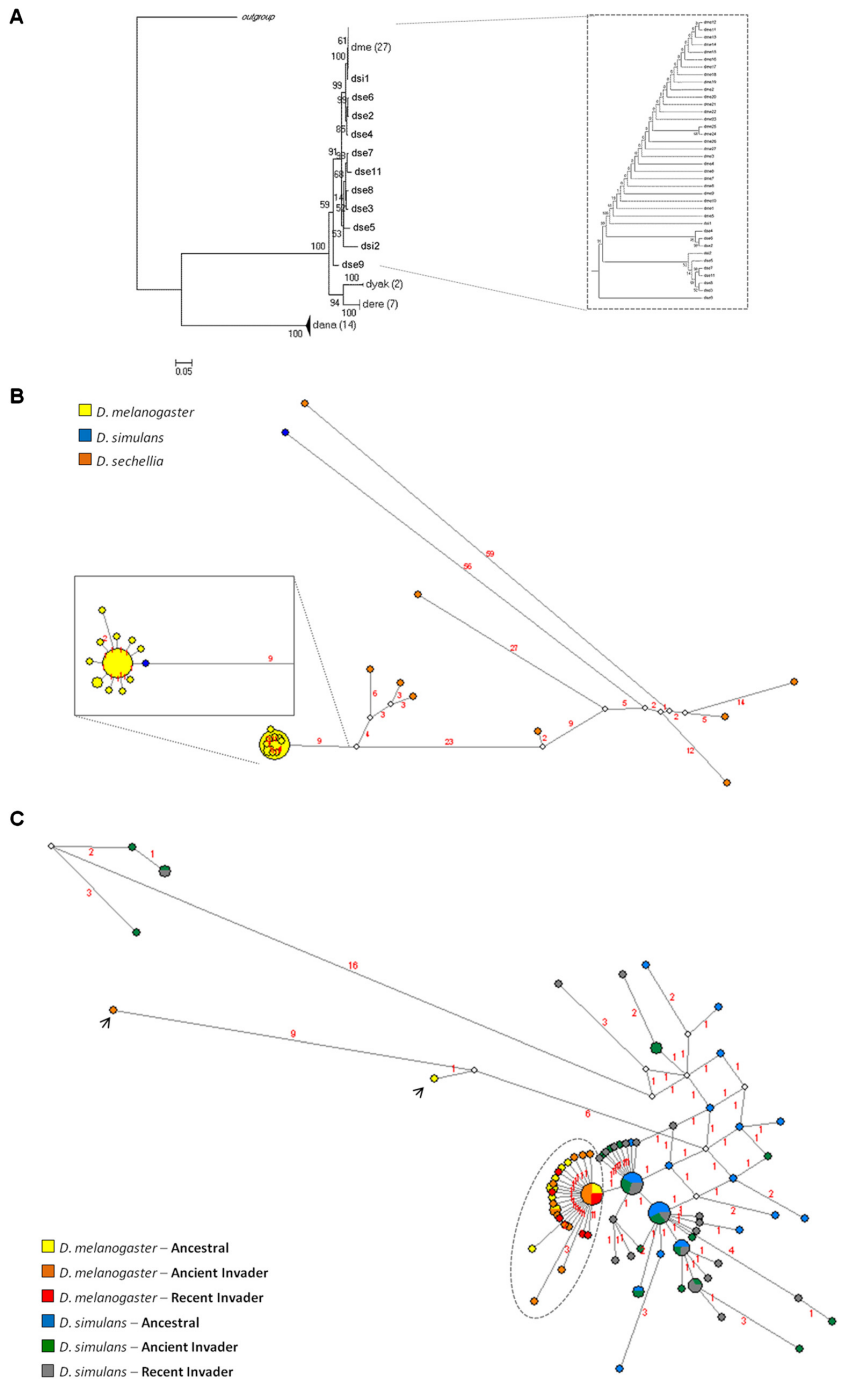
**Phylogenetic reconstructions conducted using the retrotransposon *412 *sequences in the *melanogaster *group of *Drosophila *.** (**A**) Phylogenetic analysis by maximum likelihood and (**B**) Network using the *gag* sequences of full copies obtained from sequenced genomes of the *melanogaster* group; (**C**) Network reconstructed using a region of the integrase, sequenced from samples of natural populations of *D. melanogaster* and *D. simulans*. In the network, full circles correspond to the sampled sequences; empty circles correspond to the ancestral nodes, which represent lost sequences or sequences not sampled. Circle size corresponds to sequence frequency; branch size is proportional to the number of mutations that occurred, as indicated by the numbers above branches.

The similarity of the two *D. simulans Bari1* sequences to that of *D. melanogaster* is high, with the sequences differing at only a few sites. To identify whether the proposed recent transfer is exclusive to the sequenced genomes, we sequenced a region of the transposase gene in different strains of both species and reconstructed their relationships (Figure 1C). The strains analyzed represented natural populations of different geographic origins: Africa, the ancestral site; Asia and Europe, continents that were first colonized by both species (ancient invaders); and Brazil, where colonization occurred relatively recently (recent invaders). The evolutionary reconstruction shows a sequence shared among all strains. This central sequence, which likely corresponds to the central sequence depicted in Figure 2B, could be the sequence transferred between the species. The sharing of this sequence among strains of different geographic origins (Africa, Asia, Europe and Brazil) suggests that transfer of *Bari* occurred before the global dispersal of *D. melanogaster*.

### Retrotransposon 412

As with *Bari*, we found different numbers of full-length copies (with both LTRs) of the retrotransposon *412* in different species of the *melanogaster* group. The smallest numbers were found in *D. simulans* (2) and *D. yakuba* (2), followed by *D. erecta* (8), *D. sechellia* (11), *D. ananassae* (14) and *D. melanogaster* (27*;* see Table S5 in Additional File 3). The ML phylogeny based on the *gag* region shows monophyletic branches clustering with the sequences of *D. ananassae*, *D. erecta* and *D. yakuba* with high statistical support (Figure 2A and Additional file 3: Figure S3). Like the species, the *D. ananassae 412* was the first to diverge. Next, the ancestral element of *D. yakuba* and *D. erecta* diverged, also mirroring the species divergence (Additional File 1); however, in the *melanogaster* complex, the *412* sequences did not form monophyletic groups within each species. Given the very recent divergence of *D. melanogaster, D. simulans* and *D. sechellia* (~2 and 3 Mya and 0.5 Mya, respectively [11,12,28], the sequences of *412* could not have had time to coalesce, potentially explaining the unresolved relationships among them. Alternatively, the *412* sequence could have been exchanged between these species.

To resolve the phylogenetic relationships of *412* within the *melanogaster* complex, we again used the network approach (Figure 2B). The reconstruction shows long and short branches connecting the sequences of *D. sechellia*, suggesting the presence of old and young copies in this species. These sequences are connected to two copies of *D. simulans* through median vectors (ancestral or unsampled sequences). In *D. simulans*, only two full-length sequences were sampled; these sequences are located in different regions of the network. One is related by a long branch to the old sequences of *D. sechellia* through median vectors, whereas the other is more closely related to all the sequences of *D. melanogaster*. As shown in Figure 2B, all copies of *D. melanogaster* are directly derived from this sequence of *D. simulans*, and all have short branches, indicating a very recent origin. This relationship suggests a transfer of *412* from *D. simulans* to *D. melanogaster*.

To confirm this pattern in strains derived from several natural populations, we sequenced the integrase region of the *412* element in the same strains of *D. melanogaster* and *D. simulans* analyzed for *Bari* (from Africa, Asia, Europe and South America) and reconstructed the network (Figure 2C). A similar relationship was observed in the strains of both species, where most of the *D. melanogaster* sequences show short branches and are derived from only one *D. simulans* sequence. Two sequences of *D. melanogaster* are excluded from this group. They are unresolved and connected by a median vector to the *D. simulans* sequences, as is apparent from the reticulation in the network. There are two main groups of *D. simulans* sequences: one with resolved relationships and short branches, and the other presenting reticulations and several long branches. This scenario reflects the ancient and complex evolutionary history of *412* in this species. Note that the sequence transferred to *D. melanogaster* is shared by all *D. simulans* strains, indicating that it is an active ancestral sequence in this species. In addition, all *D. melanogaster* strains, regardless of origin, share the sequence derived from *D. simulans*, which differs by only one point mutation. The sharing of ancestral sequences among strains from different continents suggests that, as with *Bari*, transfer occurred before the dispersal out of Africa.

The age of the proposed transfer was estimated using the molecular clock equation (t = *Ks*/2r). To calculate *Ks*, the 27 full-length sequences of *gag* + *pol* ORFs extracted from the sequenced *D. melanogaster* genome were compared to the ancestral sequence from *D. simulans* (Figure 2B, detail). The oldest age estimated is 146,000 y (average = 33,674 y ± 0.0084; lowest = 0.0 y), suggesting that the transfer of *412* from *D. simulans* to *D. melanogaster* occurred very recently (see Table S7 in Additional File 3). Indeed, the ages of the insertions in the *D. melanogaster* genome, as calculated by the divergence between the LTRs of each copy are 94,697 y (the highest) and 0.0 y (the lowest*;* see Figure S4 in Additional File 3). It is known that at least two factors can introduce biases into this estimation. First, the LTRs evolve, in general, faster than the coding domains of the retrotransposon sequence. Second, because the LTRs of the new copy are synthesized from only one maternal LTR at the moment of reverse transcription, the new LTRs are identical at the point of insertion. This process may conceal the accumulation of divergence between copies. Despite these biases, this approach has been widely used [29,30] and provides useful information about the date of insertion of each copy. The estimated age indicates that transposition would have started soon after the introduction of the copy and continued until very recently, congruent with empirical data and simulations [7]. The analysis shows that all full-length *412 D. melanogaster* sequences were inserted into the genome no more than 0.1 Mya, while in *D. simulans*, the insertion occurred approximately 0.3 Mya.

## Discussion

The DNA transposon *Bari* and the retrotransposon *412* are found widely in *Drosophila*, suggesting a long evolutionary history within the genus [23,31,32]. Here, we performed phylogenetic analyses involving both traditional and network approaches that allowed us to reveal the occurrence of ancestral polymorphism and recent transfer of transposable elements between *D. melanogaster* and *D. simulans*.

### Ancestral polymorphism

We performed an *in silico* search for the DNA transposon *Bari* in the sequenced genome of species of the *melanogaster* group, and we sequenced a region of the transposase in different geographic strains of *D. melanogaster* and *D. simulans*. The element exhibits structural variations related to its TIRs, which characterize various *Bari* subfamilies [23]. Both long and short TIRs were observed in the sequences analyzed in our study. The sequences of elements found in *D. ananassae* and *D. erecta*, which contain long TIRs, included stop codons and are therefore inactive, whereas the sequences of *D. melanogaster*, *D. simulans* and *D. sechellia* included short TIRs. In *D. melanogaster* and *D. simulans*, there were full-length sequences without stop codons, which therefore suggest putatively active copies. In contrast, the full-length sequences in *D. sechellia* included multiple stop codons.

The element found in *D. ananassae*, with long TIRs, is more closely related to the element of *D. melanogaster*, which has short TIRs, than to that of *D. erecta*, which also has long TIRs. These relationships produce incongruences between the element and species phylogenies. The element found in *D. erecta* (*Bari2* subfamily) is widely distributed in *Drosophila*, whereas those in *D. ananassae* (*Bari1*-like subfamily) and in the *melanogaster* complex (*Bari1* subfamily) are restricted to their respective species; this pattern indicates that the *Bari* element of *D. erecta* is older than that of *D. ananassae *[23]. Therefore, we propose that the ancestor of the *melanogaster* species group possessed at least two *Bari* variants that were stochastically inherited by the derived species. The estimated age of the common ancestor of the *Bari* sequences in the genomes of *D. ananassae* and *D. melanogaster* (~ 17 Mya) is similar to the ages of their host genes (~ 19 Mya). During this period, the proto-*melanogaster* lineage was still diversifying from its sister subgroups in Africa. The only published estimate for the diversification between the *melanogaster* and *ananassae* subgroups is ~ 44 Mya [25], but it could have occurred more recently, as all of the divergence times estimated in that study are more than two times higher than other estimates (e.g., between the *melanogaster* and *montium* subgroups, 41.3 Mya [25] and 12.7 Mya [33] and between *D. melanogaster* and *D. simulans,* 5.4 Mya [25], 2.3 Mya [33] and 2–3 Mya [11]). In conclusion, i) the phylogenetic incongruence arising from clustering the *Bari* sequences of *D. ananassae* with those of the *melanogaster* complex and ii) the older estimated age of their *Bari* ancestor with respect to the age of diversification of the *melanogaster* subgroup and the migration of its ancestral lineage to Tropical Africa support the hypothesis of vertical inheritance with stochastic retention of polymorphic sequences of *Bari* in these species. The ancestral polymorphism hypothesis is also supported by the smaller distances between the elements of the *D. ananassae* vs. *melanogaster* complex than between those of the *D. erecta* vs. *melanogaster* complex.

As described in the Background section, few reports clearly demonstrate retention of ancestral polymorphisms. One such study examines the DNA transposon *mariner*, which occurs in the *melanogaster* subgroup in *D. simulans, D. sechellia, D. mauritiana, D. yakuba* and *D. teissieri*, but not in *D. melanogaster, D. erecta* and *D. orena.* It is proposed that *mariner* was present in the ancestral species prior to the radiation of the *melanogaster* species subgroup and that the element was lost independently in the lineages leading to *D. melanogaster* and *D. orena - D. erecta.* In addition, the *mariner* sequences of *D. simulans* and *D. mauritiana* share active copies, a subset of all *mariner* sequences, that cluster together rather than according to the species phylogeny. This shared polymorphism in populations of *D. simulans* worldwide and in *D. mauritiana* indicates retention of ancestral polymorphisms [34]. In both the *mariner* study and in ours, the rates of evolution of the DNA transposon and of a host gene were compared to test the ancestral-polymorphism hypothesis and to explain incongruence between the phylogenies of the species and the transposable element.

### Transposable element recent invasion

The incongruence between the species phylogeny and the phylogenies of the *Bari* and *412* elements, along with the ages of the sequences shared between *D. melanogaster* and *D. simulans* (less than the age of the species divergence), could arise through either horizontal transfer or introgressive hybridization. An increasing number of reports in the last two decades (mostly published following the rise of large-scale genome sequencing, which allows analysis of most copies from a given genome as well as broader comparative evolutionary analysis) suggest that horizontal transfer of transposable elements occurs frequently in eukaryotes (for a review see [7]), especially in *Drosophila* (for a review see [8,9]). Particularly for *D. melanogaster* and *D. simulans*, evidence of horizontal transfer is accumulating in the literature [19,35-42], including the elements *Bari *[19,37] and *412*[19,37,42], the focus of this study; however, *D. melanogaster* and *D. simulans* can hybridize, even today, in both the laboratory [43] and in nature [44]; therefore, it is also possible to introduce transposable elements via hybridization.

In order for either horizontal transfer or introgression to occur, species must overlap in time, space and habitat. Moreover, for horizontal transfer, a shared potential vector (e.g., a virus or endobacterium) is required. Currently, the sibling species *D. melanogaster* and *D. simulans* are cosmopolitan and are sympatric in many parts of the world; however, our analyses suggest that transfer events occurred before their worldwide expansion but after species divergence. It is thought that *D. melanogaster* was the first species to diverge from a common ancestor in West Africa and that the ancestor of *D. simulans*, the proto-*simulans* lineage, migrated to east Africa and occupied the Pacific islands and then diversified. After the divergence, *D. simulans* returned to the mainland and expanded, coming into contact with *D. melanogaster* populations in Africa during the Late Pleistocene (around 120 and 9 thousand years ago) [10]; we estimate that transfer of both elements occurred during this period. Overlaps in space, time, and most likely niche, may have provided the necessary conditions for both horizontal transfer and introgression. Regardless of the precise mechanism, after the transfers occurred, both species expanded out of Africa, *D. melanogaster* with the *Homo sapiens* migration and *D. simulans* more recently, most likely during the great navigations [12].

The presence of defective fragments of *Bari* and *412* elements in the sequenced genomes of both species and the presence of two more divergent sequences of the retrotransposon *412* in populations of *D. melanogaster* (as shown in the network) indicate that both elements were present in the common ancestor of these species and were inherited by both species. The presence of full-length and putatively active copies in *D. melanogaster*, which were derived exclusively from sequences transferred from *D. simulans*, suggests that *D. melanogaster* either did not inherit active copies of both *Bari* and *412* from a common ancestor or lost these copies early in its diversification. The defective fragments that are still present today would then be remnants of the copies inherited from its ancestor. After the reintroductions of *Bari* and *412*, the transferred sequences remained active in *D. melanogaster*, giving rise to the majority of copies that are currently present in this species. We show here that the amplification of these copies occurred in a short period of time and at elevated rates, resulting in a burst of transposition. This process can be deduced from the network by the presence of identical sequences clustered in nodes or by similar sequences connected by short branches; these characteristics are observed in both species, in both the sequenced genomes and in natural populations. Bursts of transposition have been previously reported, *in silico*, for other elements in the genomes of the *melanogaster* subgroup, such as the *Helitron DINE-1* in *D. yakuba* and *D. ananassae*[45] and numerous LTR retrotransposons in the *D. melanogaster* euchromatin [20].

The element *412* occurs in two subfamilies in *D. simulans*. Only one is observed in *D. melanogaster* and is very similar to one of the subfamilies found in *D. simulans*[24,30]. Therefore, according to our data, an ancestral sequence of the *melanogaster* complex was likely inherited by both species, but diversification occurred only in *D. simulans*; later, one of the two subfamilies was transferred to *D. melanogaster*, which at the time had only defective copies derived from its ancestral lineage. The subfamilies present in *D. simulans* were the result of rearrangements, indels and point mutations in the regulatory sequences in the 5' LTR –UTR region; then, these changes gave rise to elements that were able to overcome the host control for transposition and thus able to became invaders [24,30]. This process may explain why the retrotransposon *412* remained active in *D. simulans* following its divergence and retained its capacity for amplification following its transfer to *D. melanogaster*, whose control host system, which had coevolved related with the sequences inherited from the ancestral copies, could not recognize this new element. Data from the literature (reviewed in [9]) suggest that several elements have been independently transferred between the two species over time (e.g., *Copia*, *tirant*, *Opus*, *Gypsy 2*, *Gypsy 5*, *Gypsy 6*, *297, 17.6*), but several others may have been transferred simultaneously and very recently (e.g., *412*, *Blood*, *Stalker 2*, *Transpac*, *Flea*), as can be deduced from the very similar ages of the transfers. There is a consensus that multiple elements have recently arrived in *D. melanogaster*; however, their origins either have not been suggested [20,23] or were not clearly demonstrated [38,46]. Utilization of the network approach allowed us to propose, at least for *Bari* and *412*, that *D. simulans* was the donor species.

## Conclusions

The results obtained here allowed us to propose that the incongruences observed in the phylogeny of the *Bari* and *412* elements were a result of ancestral polymorphism as well as recent invasion of *D. melanogaster* genome by these elements. The ancestral polymorphism associated with *Bari* is supported by phylogenetic incongruence, and by a divergence time of Bari between the *D. melanogaster* complex and *D. ananassae* similar to that of the host genes. The hypothesis of recent invasion of both elements is supported by phylogenetic incongruences revealed by network trees; in addition, the shortest time of divergence is found between the transposable sequences, rather than between the species involved. Moreover, *D. simulans* is thought to have transferred sequences of both elements to *D. melanogaster*. This species in turn would not have inherited or would have lost the active copy that existed in its ancestor as soon as it diverged, and all of its full-length sequences would have been derived from the sequence that was transferred from its sibling species. This introduction would have occurred in Africa before the worldwide expansion of the species, most likely in the late Pleistocene, during which *D. melanogaster* and *D. simulans* returned to sympatry in Africa after diversification in allopatry. In *D. melanogaster*, the elements would be passed through a burst of transposition, producing a high number of full-length copies over several thousand years.

## Methods

### *In silico* analyses

The search for copies of the retrotransposon *412* and of the DNA transposon *Bari* in the sequenced genomes of species of *Drosophila melanogaster* group (release 5.18 of *D. melanogaster* and 1.3 for all other species [16]) was performed using BLASTn [47]. The deposited sequences (Repbase databank [48]) described in *D. melanogaster* were used directly to search in this species. The *Bari* sequence used [GenBank: X67681] is 1,728 bp long and encodes a 340 aa transposase. The *412* sequence [GenBank: X04132] is 8,039 bp long and encodes a 452 aa *gag*-like protein and a 1,001 aa *pol*-like polyprotein (Reverse transcriptase, RNase H and Intregrase, respectively). For the other species, the deposited sequences were used to identify the reference sequence, i.e., the most complete and conserved sequence of each element in each species. These sequences were used to search for complete and incomplete copies in each genome. The complete copies were tested for the presence and integrity of the transposase gene (*Bari*) and *gag* and *pol* (*412*) using the software ORF Finder [49]. In the search, the hits with the smallest e-values (> 10^-5^) and highest RM scores (> 225) were selected. Alignment, reconstruction of the phylogeny by maximum likelihood (ML), calculation of the rate of synonymous substitutions per synonymous site (*Ks*) and analysis of the Kimura 2-parameters distance (*K2p*) [50] were performed only for full-length sequences with both TIRs and LTRs, using the package Mega5 [51]. The evolutionary relationships between sequences were also reconstructed using the package Network [52]. The ages of the transposable elements were estimated using the molecular clock equation r = k/2 T, where *r* is the rate of neutral synonymous substitution in the genus *Drosophila* (r = 0.011/site/million years [25] and *k* is the rate of divergence in the synonymous sites (*Ks*). The molecular-clock hypothesis assumes that when genes from different species are compared, the number of nucleotide changes is proportional to the speciation time. We then estimated the divergence time between species of the *melanogaster* group using the *Ks* values of the CDS of two host genes (ADH: Alcohol dehydrogenase and GAPDH: Glyceraldehyde 3 phosphate dehydrogenase 1; see Table 5S in Additional File 2) and of the *Bari* transposase. The ages of the insertions of the retrotransposon *412* were estimated using the date of divergence between both LTRs of each copy by *K2p* in the molecular clock equation.

### Molecular analyses

The phylogenetic relationships between the *Bari* sequences of strains of *D. melanogaster* and *D. simulans* of different geographic origin (Table 1) were also reconstructed. These strains were classified as ancestors (sampled in Africa) or invaders (ancient, sampled in Asia and Europe; or recent, sampled in Brazil) according to place of origin and literature reports [10].

**Table 1:** **Strains of*D. melanogaster*and*D. simulans*used in this study**

**Species**	**Classification with regard to the origin**	**Geographic Origin**	**Collector/Stock**	**GenBank Accessions**	
				***Bari-1***	**412**
*D. melanogaster*	Ancestral	Madagascar – Africa	David, J	JX140191-JX140203	JX140342-JX140346
		Congo – Africa	14021-0231.24	JX140204-JX140220	JX140347-JX140352
	Ancient Invader	Draveil – France	David, J	JX140221-JX140237	JX140353-JX140361
		Delhi – Asia	David, J	JX140238-JX140256	JX140362-JX140368
	Recent Invader	Florianopolis – Brazil	Granzotto, A	JX140257-JX140275	JX140369-JX140375
*D. simulans*	Ancestral	Madagascar – Africa	David, J	JX140276-JX140288	JX140376-JX140389
		Zimbabwe – Africa	Begun, D	JX140289-JX140299	JX140390-JX140399
	Ancient Invader	Draveil – France	Capy, P	JX140300-JX140307	JX140400-JX140411
		Gorak – New Guinea	14021-0251.009	JX140308-JX140318	JX140412-JX140420
	Recent Invader	Florianopolis – Brazil	Granzotto, A	JX140319-JX140332	JX140421-JX140435
		Pernambuco – Brazil	Rohde, C	JX140333-JX140341	JX140436-JX140445

Classification with the regard to geographic origin of the strains, name of the collectors or stock numbers and GenBank sequence accession numbers.

Genomic DNA was extracted from 50 individuals [53]. DNA concentration and integrity was analyzed via spectrophotometer (NanoDrop). Amplification (PCR) was performed using specific primers that anneals to nucleotides 412 to 1,133 (total length of 722 bp) in the *Bari* transposase gene (*Bari*_F 5’ CGG GCT GGT ATT GTT GCT AGG TTT 3’ and *Bari*_R 5’ ATC CTA CCC TTA TGG CAT GGA GCA 3’) and to nucleotides 5,622 – to 6,499 (total length of 878 bp) in the *412* integrase gene (*412*_F 5' TGG SCR AGG TCA WAR GAC AT 3’ and *412*_R 5' RCT TTS TAT STT ATA GGG CC 3’), 0.625 unit of Taq polymerase (Invitrogen), 200 ng genomic DNA, 1 mM of MgCl_2_, 1 X buffer, 0.08 mM of dNTPs and 0.4 mM of each primer, for a final volume of 25 μL. PCR conditions were as follows: initial denaturation (94°C, 120 s); followed by 30 cycles of denaturation (94°C, 45 s), annealing (69°C for *Bari* and 59°C for *412*, 45 s) and extension (72°C, 60 s). Each PCR product was analyzed by gel electrophoresis on a 1.0% agarose gel, purified (DNA GFX DNA & Gel Band, GE) and cloned (TOPO TA Cloning kit, Invitrogen) according to the manufacturer’s specifications. Approximately 30 (*D. melanogaster*) and 20 (*D. simulans*) clones were selected for plasmid extraction by phenol/chloroform protocol and sequenced using the universal primers M13F and M13R. The sequences produced were deposited in the GenBank database (Table 1).

## Competing interests

The authors declare that they have no competing interests.

## Author’s contributions

ESD conducted the bioinformatics and molecular analyses, and CMAC conceived and coordinated the project; both authors wrote the manuscript. Both authors read and approved the final manuscript.

## Supplementary Material

Additional File 1 **Figure S1: the phylogenetic relationships between species of the *melanogaster *group of *Drosophila*.** Click here for file

Additional File 2 **Tables and figure about the characteristics and evolutionary analyses of the DNA transposon *Bari *sequences found in the sequenced genomes of species of the *melanogaster *group of *Drosophila.*** Table corresponding to the *Ks* analyses of the genes *ADH* and *GAPDH*. Click here for file

Additional File 3 **Tables and figures about the characteristics and evolutionary analyses of the sequences of the retrotransposon *412 *found in the sequenced genomes of species of the *melanogaster *group of *Drosophila*.** Click here for file

## References

[B1] Lerman D Michalak P Helin A Bettencourt B Feder M Modification of heat-shock gene expression in *Drosophila melanogaster* populations via transposable elements Mol Biol Evol 2003 20 135 144 10.1093/molbev/msg015 12519916

[B2] Bejerano G Lowe C Ahituv N King B Siepel A Salama S Rubin E Kent W Haussler D A distal enhancer and an ultraconserved exon are derived from a novel retroposon Nature 2006 441 87 90 10.1038/nature04696 16625209

[B3] Feschotte C Opinion - Transposable elements and the evolution of regulatory networks Nat Rev Genet 2008 9 397 405 10.1038/nrg2337 18368054 PMC2596197

[B4] Nakayashiki H The Trickster in the genome: contribution and control of transposable elements Genes Cells 2011 16 827 841 10.1111/j.1365-2443.2011.01533.x 21722269

[B5] Almeida LM Carareto CMA Origem, Proliferação e Extinção de Elementos de Transponíveis: Qual Seria a Importância da Transferência Horizontal na Manutenção desse Ciclo? Série Monografias, SBG, Ribeirão Preto 2005 1 1 40

[B6] Capy P Langin T Anxolabehere D Bazin C Dynamics and Evolution of Transposable Elements 1998 1 Austin: Landes Bioscience

[B7] Schaack S Gilbert C Feschotte C Promiscuous DNA: horizontal transfer of transposable elements and why it matters for eukaryotic evolution Trends Ecol Evol 2010 25 537 546 10.1016/j.tree.2010.06.001 20591532 PMC2940939

[B8] Loreto ELS Carareto CMA Capy P Revisiting horizontal transfer of transposable elements in *Drosophila* Heredity 2008 100 545 554 10.1038/sj.hdy.6801094 18431403

[B9] Carareto C Tropical Africa as a cradle for horizontal transfers of transposable elements between species of the genus *Drosophila* and *Zaprionus* Mobile Genetics Elements 2011 2 179 186 10.4161/mge.1.3.18052 PMC3271551 22312591

[B10] Lachaise D Cariou ML David JR Lemeunier F Tsacas L Ashburner M Historical biogeography of the *Drosophila melanogaster* species subgroup Evolutionary Biology 1988 22 159 225

[B11] Hey J Kliman RM Population Genetics and phylogenetics of DNA sequence variation at multiple loci within the *Drosophila melanogaster* species complex Mol Biol Evol 1993 10 804 822 8355601 10.1093/oxfordjournals.molbev.a040044

[B12] Lachaise D Silvain J How two Afrotropical endemics made two cosmopolitan human commensals: the Drosophila melanogaster-D. simulans palaeogeographic riddle Genetica 2004 120 17 39 15088644 10.1023/b:gene.0000017627.27537.ef

[B13] van der Linde K Houle D A supertree analysis and literature review of the genus *Drosophila* and closely related genera (Diptera, Drosophilidae) Insect Systematics & Evolution 2008 39 241 267 10.1163/187631208788784237

[B14] Yang Y Hou Z-C Qian Y-H Kang H Zeng Q-T Increasing the data size to accurately reconstruct the phylogenetic relationships between nine subgroups of the *Drosophila melanogaster* species group (Drosophilidae, Diptera) Mol Phylogenet Evol 2012 62 214 223 10.1016/j.ympev.2011.09.018 21985965

[B15] Dobzhansky T Dreyfus A Chromosomal aberrations in Brazilian *Drosophila ananassae* Proc Natl Acad Sci 1943 29 301 305 10.1073/pnas.29.10.301 16588615 PMC1078619

[B16] Clark AG Eisen MB Smith DR Bergman CM Oliver B Markow TA Kaufman TC Kellis M Gelbart W Iyer VN Evolution of genes and genomes on the *Drosophila* phylogeny Nature 2007 450 203 218 10.1038/nature06341 17994087

[B17] Das A Population genomic and bioinformatic studies reveal evolutionary history of *Drosophila ananassae* Curr Sci 2005 89 1316 1321

[B18] Sanchez-Gracia A Maside X Charlesworth B High rate of horizontal transfer of transposable elements in *Drosophila* Trends Genet 2005 24 200 203 15797612 10.1016/j.tig.2005.02.001

[B19] Bartolome C Bello X Maside X Widespread evidence for horizontal transfer of transposable elements across *Drosophila* genomes Genome Biol 2009 10 R22 10.1186/gb-2009-10-2-r22 19226459 PMC2688281

[B20] Lerat E Burlet N Biémont C Vieira C Comparative analysis of transposable elements in the melanogaster subgroup sequenced genomes Gene 2010 473 100 109 21156200 10.1016/j.gene.2010.11.009

[B21] Caizzi R Caggese C Pimpinelli S *Bari-1*, a new transposon-like family in Drosophila melanogaster with a unique heterochromatic organization Genetics 1993 133 335 345 8382176 10.1093/genetics/133.2.335 PMC1205323

[B22] Moschetti R Caggese C Barsanti P Caizzi R Intra- and interspecies variation among *Bari-1* elements of the melanogaster species group Genetics 1998 150 239 250 9725843 10.1093/genetics/150.1.239 PMC1460315

[B23] Moschetti R Chlamydas S Marsano R Caizzi R Conserved motifs and dynamic aspects of the terminal inverted repeat organization within Bari-like transposons Mol Genet Genomics 2008 279 451 461 10.1007/s00438-008-0324-7 18247055

[B24] Mugnier N Biemont C Vieira C New regulatory regions of *Drosophila 412* retrotransposable element generated by recombination Mol Biol Evol 2005 22 747 757 15574808 10.1093/molbev/msi060

[B25] Tamura K Subramanian S Kumar S Temporal Patterns of Fruit Fly (*Drosophila*) Evolution Revealed by Mutation Clocks Mol Biol Evol 2004 21 36 44 12949132 10.1093/molbev/msg236

[B26] Posada D Crandall K Intraspecific gene genealogies: trees grafting into networks Trends Ecol Evol 2001 16 37 45 10.1016/S0169-5347(00)02026-7 11146143

[B27] Cordaux R Hedges DJ Batzer MA Retrotransposition of *Alu* elements: how many sources? Trends Genet 2004 20 464 467 10.1016/j.tig.2004.07.012 15363897

[B28] Da Lage J Kergoat G Maczkowiak F Silvain J Cariou M Lachaise D A phylogeny of Drosophilidae using the *Amyrel* gene: questioning the *Drosophila melanogaster* species group boundaries Journal of Zoological Systematics and Evolutionary Research 2007 45 47 63 10.1111/j.1439-0469.2006.00389.x

[B29] Bowen N McDonald J *Drosophila* Euchromatic LTR Retrotransposons are Much Younger Than the Host Species in Which They Reside Genome Res 2001 11 1527 1540 10.1101/gr.164201 11544196 PMC311128

[B30] Mugnier N Gueguen L Vieira C Biemont C The heterochromatic copies of the LTR retrotransposons as a record of the genomic events that have shaped the *Drosophila melanogaster* genome Gene 2008 411 87 93 10.1016/j.gene.2008.01.010 18281162

[B31] Cizeron G Lemeunier F Loevenbruck C Brehm A Biemont C Distribution of the retrotransposable element 412 in *Drosophila* species Mol Biol Evol 1998 15 1589 1599 10.1093/oxfordjournals.molbev.a025887 9866195

[B32] Caggese C Pimpinelli S Barsanti P Caizzi R The distribution of the transposable element Bari-1 in the *Drosophila melanogaster* and *Drosophila simulans* genomes Genetica 1995 96 269 283 10.1007/BF01439581 8522166

[B33] Russo C Takezaki N Nei M Molecular phylogeny and divergence times of Drosophilid species Mol Biol Evol 1995 12 391 404 7739381 10.1093/oxfordjournals.molbev.a040214

[B34] Maruyama K Hartl DL Evidence for interspecific transfer of the transposable element mariner between *Drosophila* and *Zaprionus* J Mol Evol 1991 33 514 524 10.1007/BF02102804 1664000

[B35] Bregliano JC, Kidwell MG Hybrid dysgenesis determinants 1983 New York: Academic Pres

[B36] Jordan IK Matyunina LV McDonald JF Evidence for the recent horizontal transfer of long terminal repeat retrotransposon Proc Natl Acad Sci USA 1999 96 12621 12625 10.1073/pnas.96.22.12621 10535972 PMC23018

[B37] Sanchez-Gracia A Maside X Charlesworth B High rate of horizontal transfer of transposable elements in *Drosophila* Trends Genet 2005 21 200 203 10.1016/j.tig.2005.02.001 15797612

[B38] Ludwig A Valente V Loreto ELS Multiple invasions of Errantivirus in the genus *Drosophila* Insect Mol Biol 2008 17 113 124 10.1111/j.1365-2583.2007.00787.x 18353101

[B39] Vidal NM Ludwig A Loreto ELS Evolution of *Tom, 297, 17.6* and rover retrotransposons in Drosophilidae species Mol Genet Genomics 2009 282 351 362 10.1007/s00438-009-0468-0 19585148

[B40] Depra M Valente VLD Margis R Loreto ELS The hobo transposon and hobo-related elements are expressed as developmental genes in *Drosophila* Gene 2009 448 57 63 10.1016/j.gene.2009.08.012 19720121

[B41] de Setta N Van Sluys MA Capy P Carareto CMA Multiple invasions of *Gypsy* and *Micropia* retroelements in genus *Zaprionus* and *melanogaster* subgroup of the genus *Drosophila* BMC Evol Biol 2009 9 279 10.1186/1471-2148-9-279 19954522 PMC2797524

[B42] Lerat E Burlet N Biemont C Vieira C Comparative analysis of transposable elements in the *melanogaster* subgroup sequenced genomes Gene 2011 473 100 109 10.1016/j.gene.2010.11.009 21156200

[B43] de Lucca M Carareto C Ceron C Distribution of the *Bari-I* transposable element in stable hybrid strains between *Drosophila melanogaster* and *Drosophila simulans* and in Brazilian populations of these species Genetics and Molecular Biology 2007 30 676 680 10.1590/S1415-47572007000400028

[B44] Sperlich D Hybrids between *Drosophila melanogaster* and *D. simulans* in nature Drosophila Information Service 1962 36 118

[B45] Yang HP Hung TL You TL Yang TH Genomewide comparative analysis of the highly abundant transposable element DINE-1 suggests a recent transpositional burst in *Drosophila yakuba* Genetics 2006 173 189 196 10.1534/genetics.105.051714 16387876 PMC1461449

[B46] Heredia F Loreto ELS Valente VLS Complex evolution of gypsy in drosophilid species Mol Biol Evol 2004 21 1831 1842 10.1093/molbev/msh183 15175416

[B47] Altschul SF Gish W Miller W Myers EW Lipman DJ Basic local alignment search tool J Mol Biol 1990 215 403 410 2231712 10.1016/S0022-2836(05)80360-2

[B48] Jurka J Kapitonov VV Pavlicek A Klonowski P Kohany O Walichiewicz J Repbase update, a database of eukaryotic repetitive elements Cytogenet Genome Res 2005 110 462 467 10.1159/000084979 16093699

[B49] Open Reading Frame Finderhttp://www.ncbi.nlm.nih.gov/gorf/gorf.html

[B50] Kimura M A simple method for estimating evolutionary rates of base substitutions through comparative studies of nucleotide sequences Journal of Molecular Evolution 1980 16 111 120 10.1007/BF01731581 7463489

[B51] Tamura K Peterson D Peterson N Stecher G Nei M Kumar S MEGA5: molecular evolutionary genetics analysis using maximum likelihood, evolutionary distance, and maximum parsimony methods Mol Biol Evol 2011 28 2731 2739 10.1093/molbev/msr121 21546353 PMC3203626

[B52] Bandelt H Forster P Rohl A Median-joining networks for inferring intraspecific phylogenies Mol Biol Evol 1999 16 37 48 10.1093/oxfordjournals.molbev.a026036 10331250

[B53] Jowett T Roberts DB Preparation of nucleic acids In Drosophila: A Practical Approach 1986 Oxford: IRL Press 275 285

