# Supplementary material for: Ancestral polymorphism and recent invasion of transposable elements in Drosophila species
Source: BMC Evol Biol. 2012 Jul 23;12:119. doi: 10.1186/1471-2148-12-119 (PMC3499218; doi:10.1186/1471-2148-12-119)
Supplement: Additional File 1 — Figure S1: the phylogenetic relationships between species of the melanogaster group of Drosophila. [file 1471-2148-12-119-S1.doc]

**Additional File 1 –** Tables of the characteristics of the transposon *Bari* sequences found in sequenced genomes of species of the *melanogaster* group of *Drosophila*., localization of the full-length sequences in the sequenced genomes and evolutionary analyses.

**Table S1 –** Characteristics of the transposon *Bari* sequences annotated in the sequenced genomes of *melanogaster* group species.

| **Species** | **Complete ^a^** | |  | **Incomplete** | |
| --- | --- | --- | --- | --- | --- |
|  | **n** | **Size ^b^** |  | **n** | **Size ^b^** |
| *D. melanogaster* | 11 | 1,716.4 ± 9.5 |  | 57 | 520.3 ± 52.08 |
| *D. simulans* | 2 | 1,728 ± 0 |  | 7 | 528.1 ± 95.73 |
| *D. sechellia* | 2 | 1,728 ± 1 |  | 5 | 871.8 ± 292.12 |
| *D. yakuba* | - | - |  | 1 | 215 |
| *D. erecta* | 7 | 1,690.3 ± 20.1 |  | 38 | 601.5 ± 76.33 |
| *D. ananassae* | 4 | 1,711.2 ± 21.99 |  | 31 | 704.06 ± 93.95 |

^a^ Copies with both Terminal Inverted Repeat (TIRs); ^b^ Average and standard error.

**Table S2 - Sequences of the *Bari* transposon in the sequenced genomes of subgroup *melanogaster*.**

| **Sequence** | **Insertion**  **Localization** | **Insertion**  **Orientation** | **Begin** | **End** |
| --- | --- | --- | --- | --- |
| **dme1** | 2L | - | 772243 | 770516 |
| **dme2** | 2RHet | + | 1422687 | 1424414 |
| **dme3** | 2R | - | 14747524 | 14745796 |
| **dme4** | 3R | + | 14962847 | 14964586 |
| **dme5** | 3R | - | 19385900 | 19384173 |
| **dme6** | 4 | + | 860624 | 862351 |
| **dme7** | U | - | 3230414 | 3228687 |
| **dme8** | U | + | 4473256 | 4474983 |
| **dme9** | U | + | 4474980 | 4476704 |
| **dme10** | U | + | 6565542 | 6567170 |
| **dme11** | U | + | 7168542 | 7170231 |
| **dse1** | scaffold_3 | + | 1794315 | 1796041 |
| **dse2** | scaffold_493 | + | 8176 | 6448 |
| **dsi1** | scaffold_2R | - | 7167435 | 7170162 |
| **dsi2** | X | + | 3123384 | 3126111 |
| **der1** | scaffold_4512 | + | 1132151 | 1130545 |
| **der2** | scaffold_4512 | + | 25186 | 26930 |
| **der3** | scaffold_4784 | + | 25130383 | 25132055 |
| **der4** | scaffold_4845 | + | 1047085 | 1045363 |
| **der5** | scaffold_4845 | + | 2177040 | 2178757 |
| **der6** | scaffold_4929 | + | 23035279 | 23033646 |
| **der7** | scaffold_4929 | + | 23829773 | 23831504 |
| **dan1** | scaffold_12905 | + | 319215 | 320944 |
| **dan2** | scaffold_13089 | + | 175993 | 174252 |
| **dan3** | scaffold_13099 | + | 2901122 | 2902767 |
| **dan4** | scaffold_13417 | + | 3446828 | 3448554 |

dme = *D. melanogaster*; dse = *D. sechellia*; dsi = *D. simulans*; der = *D. erecta*; dan = *D. ananassae*.


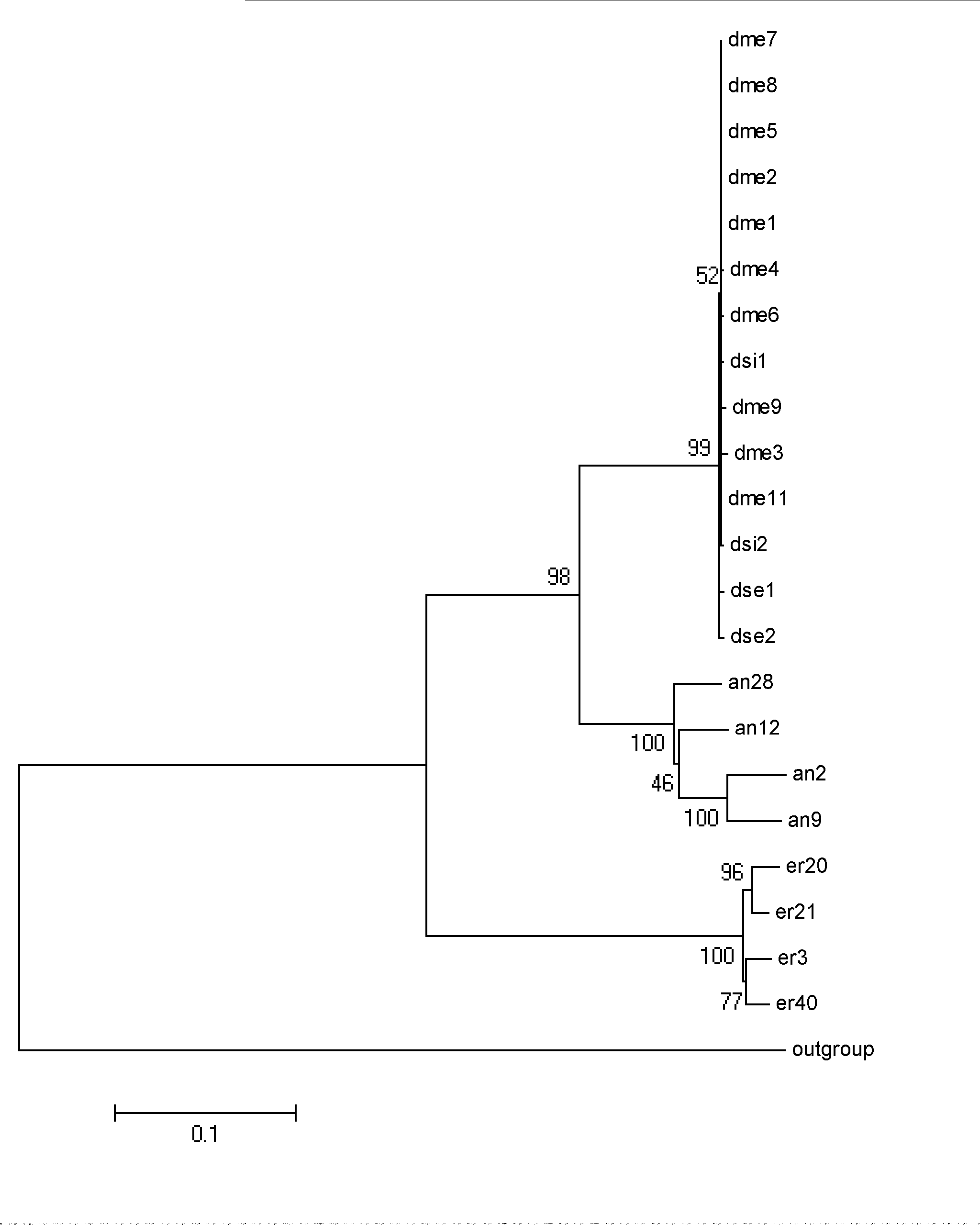


**Figure S2 - Phylogeny of full sequences of the transposon *Bari* in sequenced genome of melanogaster group.** Phylogenetic reconstruction by Maximum Likelihood using the sequences of the *gag* of the full copies obtained from genome sequenced of the *melanogaster* group.

**Table S3 – Estimates of evolutionary divergence (*K2p*) between sequences using the region of the transposase of the transposon *Bari* in species of *melanogaster* group.** Analyses were conducted using the Kimura 2-parameter model, with 22 nucleotide sequences (986 nt). All positions containing gaps and missing data were eliminated. The values of distance are show below and standard error estimate(s) above the diagonal.

|  | **1** | **2** | **3** | **4** | **5** | **6** | **7** | **8** | **9** | **10** | **11** | **12** | **13** | **14** | **15** | **16** | **17** | **18** | **19** | **20** | **21** | **22** |
| --- | --- | --- | --- | --- | --- | --- | --- | --- | --- | --- | --- | --- | --- | --- | --- | --- | --- | --- | --- | --- | --- | --- |
| **1.dme1** |  | 0.001 | 0.002 | 0.001 | 0.001 | 0.002 | 0.001 | 0.001 | 0.002 | 0.001 | 0.002 | 0.002 | 0.002 | 0.001 | 0.024 | 0.025 | 0.023 | 0.025 | 0.013 | 0.014 | 0.011 | 0.013 |
| **2.dme2** | 0.001 |  | 0.002 | 0.001 | 0 | 0.001 | 0 | 0 | 0.001 | 0 | 0.002 | 0.001 | 0.001 | 0.001 | 0.024 | 0.025 | 0.023 | 0.025 | 0.013 | 0.014 | 0.011 | 0.013 |
| **3.dme3** | 0.004 | 0.003 |  | 0.002 | 0.002 | 0.002 | 0.002 | 0.002 | 0.002 | 0.002 | 0.002 | 0.002 | 0.002 | 0.002 | 0.024 | 0.025 | 0.023 | 0.025 | 0.014 | 0.014 | 0.011 | 0.013 |
| **4.dme4** | 0.002 | 0.001 | 0.004 |  | 0.001 | 0.002 | 0.001 | 0.001 | 0.002 | 0.001 | 0.002 | 0.002 | 0.002 | 0.001 | 0.024 | 0.025 | 0.023 | 0.025 | 0.014 | 0.014 | 0.011 | 0.013 |
| **5.dme5** | 0.001 | 0 | 0.003 | 0.001 |  | 0.001 | 0 | 0 | 0.001 | 0 | 0.002 | 0.001 | 0.001 | 0.001 | 0.024 | 0.025 | 0.023 | 0.025 | 0.013 | 0.014 | 0.011 | 0.013 |
| **6.dme6** | 0.003 | 0.002 | 0.005 | 0.003 | 0.002 |  | 0.001 | 0.001 | 0.002 | 0.001 | 0.002 | 0.002 | 0.002 | 0.002 | 0.024 | 0.025 | 0.023 | 0.025 | 0.014 | 0.014 | 0.011 | 0.013 |
| **7.dme7** | 0.001 | 0 | 0.003 | 0.001 | 0 | 0.002 |  | 0 | 0.001 | 0 | 0.002 | 0.001 | 0.001 | 0.001 | 0.024 | 0.025 | 0.023 | 0.025 | 0.013 | 0.014 | 0.011 | 0.013 |
| **8.dme8** | 0.001 | 0 | 0.003 | 0.001 | 0 | 0.002 | 0 |  | 0.001 | 0 | 0.002 | 0.001 | 0.001 | 0.001 | 0.024 | 0.025 | 0.023 | 0.025 | 0.013 | 0.014 | 0.011 | 0.013 |
| **9.dme9** | 0.003 | 0.002 | 0.005 | 0.003 | 0.002 | 0.004 | 0.002 | 0.002 |  | 0.001 | 0.002 | 0.002 | 0.002 | 0.002 | 0.024 | 0.025 | 0.023 | 0.025 | 0.014 | 0.014 | 0.011 | 0.013 |
| **10.dme11** | 0.001 | 0 | 0.003 | 0.001 | 0 | 0.002 | 0 | 0 | 0.002 |  | 0.002 | 0.001 | 0.001 | 0.001 | 0.024 | 0.025 | 0.023 | 0.025 | 0.013 | 0.014 | 0.011 | 0.013 |
| **11.dse1** | 0.004 | 0.003 | 0.006 | 0.004 | 0.003 | 0.005 | 0.003 | 0.003 | 0.005 | 0.003 |  | 0.002 | 0.002 | 0.002 | 0.024 | 0.024 | 0.023 | 0.025 | 0.013 | 0.014 | 0.011 | 0.013 |
| **12.dse2** | 0.004 | 0.003 | 0.006 | 0.004 | 0.003 | 0.005 | 0.003 | 0.003 | 0.005 | 0.003 | 0.004 |  | 0.002 | 0.002 | 0.024 | 0.024 | 0.023 | 0.025 | 0.013 | 0.014 | 0.011 | 0.013 |
| **13.dsi1** | 0.003 | 0.002 | 0.005 | 0.003 | 0.002 | 0.004 | 0.002 | 0.002 | 0.004 | 0.002 | 0.005 | 0.005 |  | 0.002 | 0.024 | 0.025 | 0.023 | 0.025 | 0.013 | 0.014 | 0.011 | 0.012 |
| **14.dsi2** | 0.002 | 0.001 | 0.004 | 0.002 | 0.001 | 0.003 | 0.001 | 0.001 | 0.003 | 0.001 | 0.004 | 0.004 | 0.003 |  | 0.024 | 0.025 | 0.023 | 0.025 | 0.013 | 0.014 | 0.011 | 0.013 |
| **15.der2** | 0.374 | 0.375 | 0.378 | 0.376 | 0.375 | 0.375 | 0.375 | 0.375 | 0.374 | 0.375 | 0.376 | 0.376 | 0.378 | 0.375 |  | 0.006 | 0.005 | 0.005 | 0.024 | 0.023 | 0.021 | 0.022 |
| **16.der4** | 0.375 | 0.375 | 0.376 | 0.377 | 0.375 | 0.375 | 0.375 | 0.375 | 0.375 | 0.375 | 0.377 | 0.376 | 0.378 | 0.375 | 0.034 |  | 0.004 | 0.006 | 0.025 | 0.024 | 0.022 | 0.023 |
| **17.der5** | 0.365 | 0.365 | 0.368 | 0.367 | 0.365 | 0.365 | 0.365 | 0.365 | 0.365 | 0.365 | 0.367 | 0.366 | 0.368 | 0.365 | 0.030 | 0.023 |  | 0.005 | 0.024 | 0.023 | 0.021 | 0.022 |
| **18.der7** | 0.366 | 0.366 | 0.369 | 0.368 | 0.366 | 0.366 | 0.366 | 0.366 | 0.366 | 0.366 | 0.368 | 0.368 | 0.369 | 0.366 | 0.029 | 0.034 | 0.030 |  | 0.026 | 0.025 | 0.023 | 0.024 |
| **19.dan1** | 0.186 | 0.185 | 0.187 | 0.186 | 0.185 | 0.187 | 0.185 | 0.185 | 0.187 | 0.185 | 0.187 | 0.187 | 0.187 | 0.185 | 0.390 | 0.401 | 0.393 | 0.398 |  | 0.007 | 0.008 | 0.009 |
| **20.dan2** | 0.184 | 0.182 | 0.185 | 0.184 | 0.182 | 0.185 | 0.182 | 0.182 | 0.185 | 0.182 | 0.185 | 0.185 | 0.185 | 0.182 | 0.393 | 0.402 | 0.393 | 0.398 | 0.058 |  | 0.008 | 0.009 |
| **21.dan3** | 0.160 | 0.158 | 0.161 | 0.160 | 0.158 | 0.161 | 0.158 | 0.158 | 0.161 | 0.158 | 0.160 | 0.160 | 0.161 | 0.157 | 0.366 | 0.365 | 0.352 | 0.364 | 0.078 | 0.077 |  | 0.008 |
| **22.dan4** | 0.160 | 0.158 | 0.161 | 0.160 | 0.158 | 0.160 | 0.158 | 0.158 | 0.161 | 0.158 | 0.160 | 0.160 | 0.161 | 0.158 | 0.370 | 0.373 | 0.363 | 0.369 | 0.084 | 0.083 | 0.056 |  |

dme = *D. melanogaster*; dse = *D. sechellia*; dsi = *D. simulans*; der = *D. erecta*; dan = *D. ananassae*.

**Table S4 - Estimates of evolutionary divergence (*Ks*) between sequences using the region of the transposase of the transposon *Bari* in species of *melanogaster* group.** Analyses were conducted using the Nei-Gojobori model. The analysis involved 16 nucleotide sequences (330 positions). All positions containing gaps, missing data and stop codons were eliminated. The number of synonymous substitutions per synonymous site from between sequences is shown below and standard error estimate(s) above the diagonal.

|  | **1** | **2** | **3** | **4** | **5** | **6** | **7** | **8** | **9** | **10** | **11** | **12** | **13** | **14** | **15** | **16** |
| --- | --- | --- | --- | --- | --- | --- | --- | --- | --- | --- | --- | --- | --- | --- | --- | --- |
| **1.dme1** |  | 0 | 0 | 0 | 0 | 0.005 | 0 | 0 | 0 | 0 | 0 | 0 | 0.051 | 0.053 | 0.049 | 0.049 |
| **2.dme2** | 0 |  | 0 | 0 | 0 | 0.005 | 0 | 0 | 0 | 0 | 0 | 0 | 0.051 | 0.053 | 0.049 | 0.049 |
| **3.dme3** | 0 | 0 |  | 0 | 0 | 0.005 | 0 | 0 | 0 | 0 | 0 | 0 | 0.051 | 0.053 | 0.049 | 0.048 |
| **4.dme4** | 0 | 0 | 0 |  | 0 | 0.005 | 0 | 0 | 0 | 0 | 0 | 0 | 0.051 | 0.053 | 0.049 | 0.049 |
| **5.dme5** | 0 | 0 | 0 | 0 |  | 0.005 | 0 | 0 | 0 | 0 | 0 | 0 | 0.051 | 0.053 | 0.049 | 0.049 |
| **6.dme6** | 0.004 | 0.004 | 0.004 | 0.004 | 0.004 |  | 0.005 | 0.005 | 0.005 | 0.005 | 0.005 | 0.005 | 0.053 | 0.055 | 0.051 | 0.050 |
| **7.dme7** | 0 | 0 | 0 | 0 | 0 | 0.004 |  | 0 | 0 | 0 | 0 | 0 | 0.051 | 0.053 | 0.049 | 0.049 |
| **8.dme8** | 0 | 0 | 0 | 0 | 0 | 0.004 | 0 |  | 0 | 0 | 0 | 0 | 0.051 | 0.053 | 0.049 | 0.049 |
| **9.dme9** | 0 | 0 | 0 | 0 | 0 | 0.004 | 0 | 0 |  | 0 | 0 | 0 | 0.051 | 0.053 | 0.049 | 0.049 |
| **10.dme11** | 0 | 0 | 0 | 0 | 0 | 0.004 | 0 | 0 | 0 |  | 0 | 0 | 0.051 | 0.053 | 0.049 | 0.049 |
| **11.dsi1** | 0 | 0 | 0 | 0 | 0 | 0.004 | 0 | 0 | 0 | 0 |  | 0 | 0.051 | 0.053 | 0.049 | 0.049 |
| **12.dsi2** | 0 | 0 | 0 | 0 | 0 | 0.004 | 0 | 0 | 0 | 0 | 0 |  | 0.051 | 0.053 | 0.049 | 0.049 |
| **13.dan1** | 0.419 | 0.418 | 0.421 | 0.418 | 0.418 | 0.427 | 0.418 | 0.418 | 0.419 | 0.418 | 0.418 | 0.418 |  | 0.017 | 0.019 | 0.022 |
| **14.dan2** | 0.423 | 0.423 | 0.425 | 0.423 | 0.423 | 0.431 | 0.423 | 0.423 | 0.424 | 0.423 | 0.423 | 0.423 | 0.070 |  | 0.021 | 0.020 |
| **15.dan3** | 0.386 | 0.386 | 0.384 | 0.386 | 0.386 | 0.394 | 0.386 | 0.386 | 0.386 | 0.386 | 0.386 | 0.386 | 0.082 | 0.094 |  | 0.015 |
| **16.dan4** | 0.405 | 0.405 | 0.403 | 0.405 | 0.405 | 0.413 | 0.405 | 0.405 | 0.405 | 0.405 | 0.408 | 0.405 | 0.078 | 0.088 | 0.055 |  |

dme = *D. melanogaster*; dsi = *D. simulans*; dan = *D. ananassae*.

**Table S5** - **Estimates of evolutionary divergence (*Ks*) between coding sequences of the *ADH* (Alcohol dehydrogenase) and *GAPDH* (Glyceraldehyde 3 phosphate dehydrogenase 1) genes between *D. melanogaster*, *D. simulans* and *D. ananassae* and time of divergence since the common ancestor sequence.** Analyses were conducted using the Nei-Gojobori model. All positions containing gaps, missing data and stop codons were eliminated.

| **Gene** | **Comparisons** | ***Ks*** | **Standard**  **error** | **Time**  **(My)** | **Size**  **(aa)** | **GenBank Access Number** | | |
| --- | --- | --- | --- | --- | --- | --- | --- | --- |
|  |  |  |  |  |  | ***D. ananassae*** | ***D. melanogaster*** | ***D. simulans*** |
| ***ADH*** | *D. ana* vs. *D. mel* | 0.36959 | 0.0608 | 16.800 | 256 | XM_001961486 | NM_001032098 | M36581.1 |
|  | *D. ana* vs. *D. mel* | 0.40388 | 0.0562 | 18.358 |  |  |  |  |
| ***GAPDH*** | *D. ana* vs. *D. mel* | 0.48712 | 0.0551 | 22.142 | 350 | XM_001962674.1 | NM_057219.3 | XM_002078253.1 |
|  | *D. ana* vs. *D. sim* | 0.44124 | 0.0532 | 20.056 |  |  |  |  |
|  |  |  |  |  |  |  |  |  |
| **Mean** | *D. ana* vs. *D. mel* | 0.428355 | 0.0579 | 19.471 |  |  |  |  |
|  | *D. ana* vs. *D. sim* | 0.422560 | 0.0547 | 19.207 |  |  |  |  |

*D. mel* = *D. melanogaster*; *D. mel* = *D. simulans*; *D. ana* = *D. ananassae*.
