# Supplementary material for: Ancestral polymorphism and recent invasion of transposable elements in Drosophila species
Source: BMC Evol Biol. 2012 Jul 23;12:119. doi: 10.1186/1471-2148-12-119 (PMC3499218; doi:10.1186/1471-2148-12-119)
Supplement: Additional File 2 — Tables and figure about the characteristics and evolutionary analyses of the DNA transposon Bari sequences found in the sequenced genomes of species of the melanogaster group of Drosophila. Table corresponding to the Ks analyses of the genes ADH and GAPDH. [file 1471-2148-12-119-S2.doc]

Additional File 2 – Phylogenetic relationships between the species of the *melanogaster* group of the genus *Drosophila*.


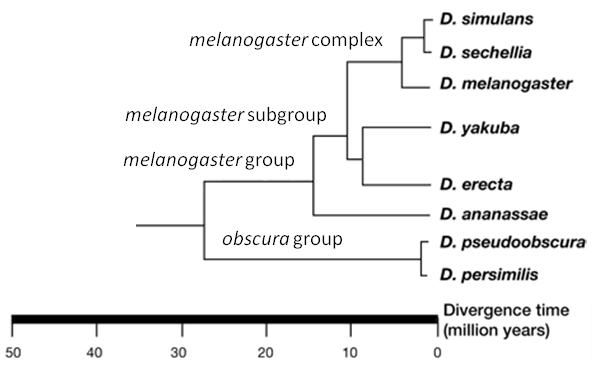


**Figure S1** – Phylogeny of the species of the *melanogaster* group. Modified from: <http://rana.lbl.gov/drosophila/>.
