# Supplementary material for: Ancestral polymorphism and recent invasion of transposable elements in Drosophila species
Source: BMC Evol Biol. 2012 Jul 23;12:119. doi: 10.1186/1471-2148-12-119 (PMC3499218; doi:10.1186/1471-2148-12-119)
Supplement: Additional File 3 — Tables and figures about the characteristics and evolutionary analyses of the sequences of the retrotransposon 412 found in the sequenced genomes of species of the melanogaster group of Drosophila. [file 1471-2148-12-119-S3.doc]

**Additional File 3 –** Tables and figures presenting the characteristics and evolutionary analyses of the sequences of the retrotransposon *412* found in the sequenced genomes of species of the *melanogaster* group of *Drosophila*.

**Table S5 –** Characteristics of the retrotransposon *412* sequences annotated in the sequenced genomes of *melanogaster* group species.

| **Species** | **Full-length ^a^** | |  | **Incompletes** | |
| --- | --- | --- | --- | --- | --- |
|  | **n** | **Size ^b^** |  | **n** | **Size ^b^** |
| *D. melanogaster* | 27 | 7,487.3 ± 28.72 |  | 37 | 977.32 ± 127.07 |
| *D. simulans* | 2 | 7,497.0 ± 72 |  | 113 | 1,335.69 ± 115.98 |
| *D. sechellia* | 5 | 7,341.4 ± 86.39 |  | 153 | 2,758.44 ± 163.82 |
| *D. yakuba* | 2 | 6,910.5 ± 114.5 |  | 44 | 1,835.09 ± 249.64 |
| *D. erecta* | 7 | 7,336.6 ± 58.28 |  | 74 | 831.36 ± 179.58 |
| *D. ananassae* | 14 | 8,395.1 ± 219.40 |  | 198 | 1,893.87 ± 107.71 |

^a^ Copies with both Long Terminal Repeats (LTRs); ^b^ Average and standard error.

**Table S6 -** Full-length **s**equences of the retrotransposon *412* in the sequenced genomes of species of the *melanogaster* group of *Drosophila*.

| **Sequence** | **Insertion**  **Localization** | **Insertion**  **Orientation** | **Begin** | **End** | **Size** |
| --- | --- | --- | --- | --- | --- |
| **dse1** | scaffold_221 | - | 21711 | 14297 | 7415 |
| **dse2** | scaffold_254 | + | 3491 | 10688 | 7198 |
| **dse3** | scaffold_111 | - | 48934 | 41520 | 7414 |
| **dse4** | scaffold_503 | + | 1432 | 8526 | 7095 |
| **dse5** | scaffold_98 | - | 36842 | 29472 | 7371 |
| **dse6** | scaffold_157 | + | 19278 | 25730 | 6453 |
| **dse7** | scaffold_309 | - | 10047 | 526 | 9522 |
| **dse8** | scaffold_119 | - | 17182 | 9628 | 7555 |
| **dse9** | scaffold_477 | - | 8890 | 718 | 8173 |
| **dse10** | scaffold_115 | + | 81260 | 87497 | 6238 |
| **dse11** | scaffold_134 | - | 8756 | 1269 | 7488 |
| **dsi1** | chrU_M_981 | - | 9360 | 1792 | 7569 |
| **dsi2** | chrU_M_1537 | + | 18546 | 25970 | 7425 |
| **dme1** | cr_2R | - | 19424670 | 19417152 | 7519 |
| **dme2** | cr_2R | - | 19809443 | 19801877 | 7567 |
| **dme3** | cr_2R | - | 20042166 | 20034646 | 7521 |
| **dme4** | cr_2R | - | 20072241 | 20064814 | 7428 |
| **dme5** | cr_2R | - | 20542666 | 20535167 | 7500 |
| **dme6** | cr_2RHet | - | 2583506 | 2576017 | 7490 |
| **dme7** | cr_3L | - | 1055161 | 1047496 | 7666 |
| **dme8** | cr_3L | + | 1219646 | 1227237 | 7592 |
| **dme9** | cr_3L | + | 4328787 | 4336227 | 7441 |
| **dme10** | cr_3L | + | 9070082 | 9077584 | 7503 |
| **dme11** | cr_3L | + | 9526908 | 9534410 | 7503 |
| **dme12** | cr_3L | + | 11557936 | 11565503 | 7568 |
| **dme13** | cr_3L | - | 17517635 | 17510135 | 7501 |
| **dme14** | cr_3L | - | 19820946 | 19813441 | 7506 |
| **dme15** | cr_3L | + | 21236900 | 21244405 | 7506 |
| **dme16** | cr_3R | - | 10935472 | 10928275 | 7198 |
| **dme17** | cr_3R | - | 11434405 | 11426941 | 7465 |
| **dme18** | cr_3R | + | 22966292 | 22973844 | 7553 |
| **dme19** | cr_3R | + | 26201316 | 26208883 | 7568 |
| **dme20** | cr_U | - | 930842 | 923349 | 7494 |
| **dme21** | cr_X | - | 197508 | 189937 | 7572 |
| **dme22** | cr_X | + | 445510 | 453014 | 7505 |

**Table S6 –** Continuation.

| **dme23** | cr_X | + | 927440 | 935015 | 7576 |
| --- | --- | --- | --- | --- | --- |
| **dme24** | cr_X | - | 3888526 | 3880951 | 7576 |
| **dme25** | cr_X | + | 10032303 | 10039743 | 7441 |
| **dme26** | cr_X | + | 19030640 | 19037501 | 6862 |
| **dme27** | cr_X | - | 21519806 | 21512270 | 7537 |
| **dere1** | scaffold_4690 | - | 18034061 | 18026620 | 7442 |
| **dere2** | scaffold_4784 | + | 23710374 | 23717661 | 7288 |
| **dere3** | scaffold_4845 | - | 1478848 | 1471528 | 7321 |
| **dere4** | scaffold_4855 | - | 7815 | 397 | 7419 |
| **dere5** | scaffold_4929 | + | 24482821 | 24490255 | 7435 |
| **dere6** | scaffold_4929 | + | 24481199 | 24488633 | 7435 |
| **dere7** | scaffold_4929 | + | 25331855 | 25338870 | 7016 |
| **dere8** | scaffold_4929 | + | 25338459 | 25345893 | 7435 |
| **dyak1** | v2_chr2h_random_26 | + | 383531 | 390555 | 7025 |
| **dyak2** | v2_chrUn_258 | - | 8548 | 1753 | 6796 |
| **dana1** | scaffold_10225 | + | 46671 | 54808 | 8138 |
| **dana2** | scaffold_12586 | + | 144426 | 152534 | 8109 |
| **dana3** | scaffold_12911 | + | 489570 | 497641 | 8071 |
| **dana4** | scaffold_12911 | + | 570181 | 578190 | 8010 |
| **dana5** | scaffold_13010 | - | 137803 | 128829 | 8975 |
| **dana6** | scaffold_13010 | + | 354265 | 362357 | 8093 |
| **dana7** | scaffold_13034 | + | 688362 | 696458 | 8097 |
| **dana8** | scaffold_13079 | + | 336999 | 344964 | 7965 |
| **dana9** | scaffold_13230 | + | 1879109 | 1890167 | 11059 |
| **dana10** | scaffold_13250 | + | 1879278 | 1887386 | 8109 |
| **dana11** | scaffold_13260 | - | 518146 | 510042 | 8105 |
| **dana12** | scaffold_13277 | + | 95272 | 104014 | 8743 |
| **dana13** | scaffold_13339 | - | 2088136 | 2080050 | 8087 |
| **dana14** | scaffold_13417 | - | 4473039 | 4465069 | 7971 |


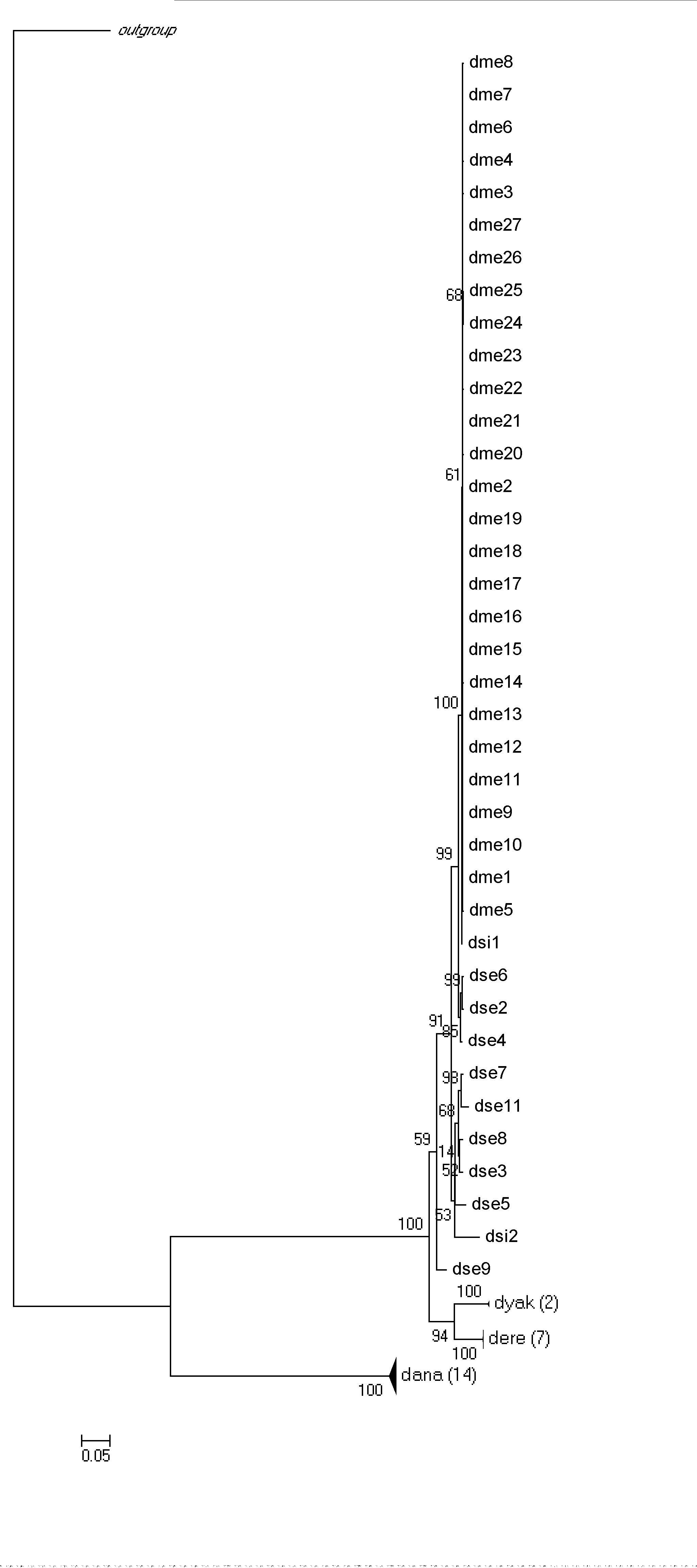


Figure S3 – Evolutionary relationships between the full-length sequences of the retrotransposon *412* in sequenced genomes of species of the *melanogaster* group of *Drosophila*. Phylogenetic reconstruction by Maximum Likelihood using the *gag* sequences.

**Table S7 - Evolutionary divergence (*Ks*) between full-length sequences of the retrotransposon *412* in *D. melanogaster* and *D. simulans* using the *gag* plus *pol* region.** Analyses were conducted using the Nei-Gojobori model. The analysis involved 27 nucleotide sequences (1,443 positions). All positions containing gaps, missing data and stop codon were eliminated. The number of synonymous substitutions per synonymous site from between sequences is shown below and standard error estimate(s) above the diagonal.

|  | **1** | **2** | **3** | **4** | **5** | **6** | **7** | **8** | **9** | **10** | **11** | **12** | **13** | **14** | **15** | **16** | **17** | **18** | **19** | **20** | **21** | **22** | **23** | **24** | **25** | **26** | **27** |
| --- | --- | --- | --- | --- | --- | --- | --- | --- | --- | --- | --- | --- | --- | --- | --- | --- | --- | --- | --- | --- | --- | --- | --- | --- | --- | --- | --- |
| **1.dsi1** |  | 0 | 0.001 | 0.002 | 0.001 | 0.001 | 0.001 | 0.001 | 0 | 0 | 0.001 | 0.001 | 0 | 0 | 0 | 0 | 0.001 | 0 | 0 | 0.002 | 0.002 | 0 | 0 | 0 | 0 | 0 | 0.001 |
| **2.dme6** | 0 |  | 0.001 | 0.002 | 0.001 | 0.001 | 0.001 | 0.001 | 0 | 0 | 0.001 | 0.001 | 0 | 0 | 0 | 0 | 0.001 | 0 | 0 | 0.002 | 0.002 | 0 | 0 | 0 | 0 | 0 | 0.001 |
| **3.dme1** | 0.002 | 0.002 |  | 0.002 | 0.001 | 0.001 | 0.002 | 0.002 | 0.001 | 0.001 | 0.002 | 0.002 | 0.001 | 0.001 | 0.001 | 0.001 | 0 | 0.001 | 0.001 | 0.002 | 0.002 | 0.001 | 0.001 | 0.001 | 0.001 | 0.001 | 0.001 |
| **4.dme2** | 0.002 | 0.002 | 0.004 |  | 0.002 | 0.002 | 0.002 | 0.002 | 0.002 | 0.002 | 0.002 | 0.002 | 0.002 | 0.002 | 0.002 | 0.002 | 0.002 | 0.002 | 0.002 | 0.002 | 0.002 | 0.002 | 0.002 | 0.002 | 0.002 | 0.002 | 0.002 |
| **5.dme3** | 0.001 | 0.001 | 0.001 | 0.003 |  | 0 | 0.002 | 0.002 | 0.001 | 0.001 | 0.001 | 0.001 | 0.001 | 0.001 | 0.001 | 0.001 | 0.001 | 0.001 | 0.001 | 0.001 | 0.001 | 0.001 | 0.001 | 0.001 | 0.001 | 0.001 | 0 |
| **6.dme4** | 0.001 | 0.001 | 0.001 | 0.003 | 0 |  | 0.002 | 0.002 | 0.001 | 0.001 | 0.001 | 0.001 | 0.001 | 0.001 | 0.001 | 0.001 | 0.001 | 0.001 | 0.001 | 0.001 | 0.001 | 0.001 | 0.001 | 0.001 | 0.001 | 0.001 | 0 |
| **7.dme5** | 0.001 | 0.001 | 0.003 | 0.003 | 0.002 | 0.002 |  | 0.002 | 0.001 | 0.001 | 0.001 | 0.001 | 0.001 | 0.001 | 0.001 | 0.001 | 0.002 | 0.001 | 0.001 | 0.002 | 0.002 | 0.001 | 0.001 | 0.001 | 0.001 | 0.001 | 0.002 |
| **8.dme7** | 0.001 | 0.001 | 0.003 | 0.003 | 0.002 | 0.002 | 0.002 |  | 0.001 | 0.001 | 0.002 | 0.002 | 0.001 | 0.001 | 0.001 | 0.001 | 0.002 | 0.001 | 0.001 | 0.001 | 0.002 | 0.001 | 0.001 | 0.001 | 0.001 | 0.001 | 0.002 |
| **9.dme12** | 0 | 0 | 0.002 | 0.002 | 0.001 | 0.001 | 0.001 | 0.001 |  | 0 | 0.001 | 0.001 | 0 | 0 | 0 | 0 | 0.001 | 0 | 0 | 0.002 | 0.002 | 0 | 0 | 0 | 0 | 0 | 0.001 |
| **10.dme8** | 0 | 0 | 0.002 | 0.002 | 0.001 | 0.001 | 0.001 | 0.001 | 0 |  | 0.001 | 0.001 | 0 | 0 | 0 | 0 | 0.001 | 0 | 0 | 0.002 | 0.002 | 0 | 0 | 0 | 0 | 0 | 0.001 |
| **11.dme13** | 0.001 | 0.001 | 0.003 | 0.003 | 0.002 | 0.002 | 0.002 | 0.002 | 0.001 | 0.001 |  | 0.001 | 0.001 | 0.001 | 0.001 | 0.001 | 0.002 | 0.001 | 0.001 | 0.002 | 0.002 | 0.001 | 0.001 | 0.001 | 0.001 | 0.001 | 0.001 |
| **12.dme14** | 0.001 | 0.001 | 0.003 | 0.003 | 0.002 | 0.002 | 0.002 | 0.002 | 0.001 | 0.001 | 0.002 |  | 0.001 | 0.001 | 0.001 | 0.001 | 0.002 | 0.001 | 0.001 | 0.002 | 0.002 | 0.001 | 0.001 | 0.001 | 0.001 | 0.001 | 0.001 |
| **13.dme15** | 0 | 0 | 0.002 | 0.002 | 0.001 | 0.001 | 0.001 | 0.001 | 0 | 0 | 0.001 | 0.001 |  | 0 | 0 | 0 | 0.001 | 0 | 0 | 0.002 | 0.002 | 0 | 0 | 0 | 0 | 0 | 0.001 |
| **14.dme9** | 0 | 0 | 0.002 | 0.002 | 0.001 | 0.001 | 0.001 | 0.001 | 0 | 0 | 0.001 | 0.001 | 0 |  | 0 | 0 | 0.001 | 0 | 0 | 0.002 | 0.002 | 0 | 0 | 0 | 0 | 0 | 0.001 |
| **15.dme10** | 0 | 0 | 0.002 | 0.002 | 0.001 | 0.001 | 0.001 | 0.001 | 0 | 0 | 0.001 | 0.001 | 0 | 0 |  | 0 | 0.001 | 0 | 0 | 0.002 | 0.002 | 0 | 0 | 0 | 0 | 0 | 0.001 |
| **16.dme11** | 0 | 0 | 0.002 | 0.002 | 0.001 | 0.001 | 0.001 | 0.001 | 0 | 0 | 0.001 | 0.001 | 0 | 0 | 0 |  | 0.001 | 0 | 0 | 0.002 | 0.002 | 0 | 0 | 0 | 0 | 0 | 0.001 |
| **17.dme16** | 0.002 | 0.002 | 0 | 0.004 | 0.001 | 0.001 | 0.003 | 0.003 | 0.002 | 0.002 | 0.003 | 0.003 | 0.002 | 0.002 | 0.002 | 0.002 |  | 0.001 | 0.001 | 0.002 | 0.002 | 0.001 | 0.001 | 0.001 | 0.001 | 0.001 | 0.001 |
| **18.dme17** | 0 | 0 | 0.002 | 0.002 | 0.001 | 0.001 | 0.001 | 0.001 | 0 | 0 | 0.001 | 0.001 | 0 | 0 | 0 | 0 | 0.002 |  | 0 | 0.002 | 0.002 | 0 | 0 | 0 | 0 | 0 | 0.001 |
| **19.dme18** | 0 | 0 | 0.002 | 0.002 | 0.001 | 0.001 | 0.001 | 0.001 | 0 | 0 | 0.001 | 0.001 | 0 | 0 | 0 | 0 | 0.002 | 0 |  | 0.002 | 0.002 | 0 | 0 | 0 | 0 | 0 | 0.001 |
| **20.dme19** | 0.002 | 0.002 | 0.002 | 0.004 | 0.001 | 0.001 | 0.003 | 0.001 | 0.002 | 0.002 | 0.003 | 0.003 | 0.002 | 0.002 | 0.002 | 0.002 | 0.002 | 0.002 | 0.002 |  | 0.002 | 0.002 | 0.002 | 0.002 | 0.002 | 0.002 | 0.001 |
| **21.dme20** | 0.003 | 0.003 | 0.003 | 0.005 | 0.002 | 0.002 | 0.004 | 0.004 | 0.003 | 0.003 | 0.004 | 0.004 | 0.003 | 0.003 | 0.003 | 0.003 | 0.003 | 0.003 | 0.003 | 0.003 |  | 0.002 | 0.002 | 0.002 | 0.002 | 0.002 | 0.001 |
| **22.dme25** | 0 | 0 | 0.002 | 0.002 | 0.001 | 0.001 | 0.001 | 0.001 | 0 | 0 | 0.001 | 0.001 | 0 | 0 | 0 | 0 | 0.002 | 0 | 0 | 0.002 | 0.003 |  | 0 | 0 | 0 | 0 | 0.001 |
| **23.dme21** | 0 | 0 | 0.002 | 0.002 | 0.001 | 0.001 | 0.001 | 0.001 | 0 | 0 | 0.001 | 0.001 | 0 | 0 | 0 | 0 | 0.002 | 0 | 0 | 0.002 | 0.003 | 0 |  | 0 | 0 | 0 | 0.001 |
| **24.dme27** | 0 | 0 | 0.002 | 0.002 | 0.001 | 0.001 | 0.001 | 0.001 | 0 | 0 | 0.001 | 0.001 | 0 | 0 | 0 | 0 | 0.002 | 0 | 0 | 0.002 | 0.003 | 0 | 0 |  | 0 | 0 | 0.001 |
| **25.dme24** | 0 | 0 | 0.002 | 0.002 | 0.001 | 0.001 | 0.001 | 0.001 | 0 | 0 | 0.001 | 0.001 | 0 | 0 | 0 | 0 | 0.002 | 0 | 0 | 0.002 | 0.003 | 0 | 0 | 0 |  | 0 | 0.001 |
| **26.dme22** | 0 | 0 | 0.002 | 0.002 | 0.001 | 0.001 | 0.001 | 0.001 | 0 | 0 | 0.001 | 0.001 | 0 | 0 | 0 | 0 | 0.002 | 0 | 0 | 0.002 | 0.003 | 0 | 0 | 0 | 0 |  | 0.001 |
| **27.dme23** | 0.001 | 0.001 | 0.001 | 0.003 | 0 | 0 | 0.002 | 0.002 | 0.001 | 0.001 | 0.002 | 0.002 | 0.001 | 0.001 | 0.001 | 0.001 | 0.001 | 0.001 | 0.001 | 0.001 | 0.002 | 0.001 | 0.001 | 0.001 | 0.001 | 0.001 |  |

dme = *D. melanogaster*; dsi = *D. simulans*.


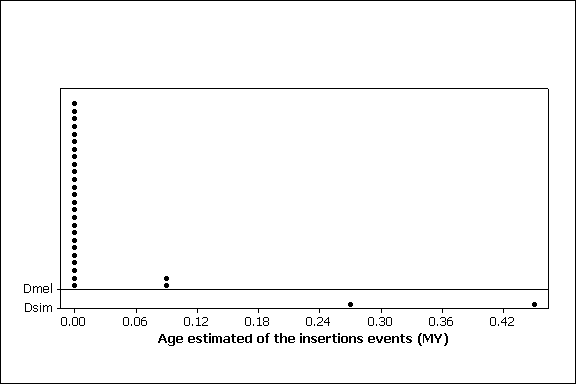


**Figure S4 –** Age at insertion of the full-length copies of the retrotransposon *412* in *D. melanogaster* and *D. simulans*.
